# Biased cognition in East Asian and Western cultures

**DOI:** 10.1371/journal.pone.0223358

**Published:** 2019-10-15

**Authors:** Jenny Yiend, Julia André, Louise Smith, Lu Hua Chen, Timothea Toulopoulou, Eric Chen, Pak Sham, Brian Parkinson

**Affiliations:** 1 King’s College London, London, England, United Kingdom; 2 Department of Psychiatry, University of Hong Kong, Hong Kong; 3 Department of Psychology, University of Hong Kong, Hong Kong; 4 Department of Experimental Psychology, University of Oxford, Oxford, England, United Kingdom; Ghent University, BELGIUM

## Abstract

The majority of cognitive bias research has been conducted in Western cultures. We examined cross-cultural differences in cognitive biases, comparing Westerners’ and East Asians’ performance and acculturation following migration to the opposite culture. Two local (UK, Hong Kong) and four migrant (short-term and long-term migrants to each culture) samples completed culturally validated tasks measuring attentional and interpretation bias. Hong Kong residents were more positively biased than people living in the UK on several measures, consistent with the lower prevalence of psychological disorders in East Asia. Migrants to the UK had reduced positive biases on some tasks, while migrants to Hong Kong were more positive, compared to their respective home counterparts, consistent with acculturation in attention and interpretation biases. These data illustrate the importance of cultural validation of findings and, if replicated, would have implications for the mental health and well-being of migrants.

## Introduction

Cognitive biases occur when one type of information is consistently favored for further processing over other types of information [1], [2]. The majority of cognitive bias research has been conducted in Western cultures and none that we know of has directly compared the profile of biases across different cultures as they manifest in the general population or considered the effects of migration upon biased cognitive function (i.e. acculturation of biases). We report a preliminary investigation of cultural differences in bias and acculturation of biases by testing for a difference in bias between healthy East Asian and Western samples. In an increasingly globalized world migration might play an important role in normalizing any cultural differences and we therefore also examined whether migration alters bias. Specifically, we examined cultural differences by comparing healthy United Kingdom (UK) and Hong Kong (HK) participants. We examined acculturation by comparing UK migrants to HK and HK migrants to the UK with their non-migrant counterparts.

An important context for the present study is the broader literature suggesting that positive biases are associated with psychological well-being, and negative biases are associated with psychopathology. Although we did not directly investigate these links in the present study, they could have important implications for our cross-cultural findings. Therefore we now briefly review the literature related to each association and the extent to which these links replicate in non-western cultures.

Positive cognitive biases (i.e. preferential processing of positive over negative or neutral information) have been studied in relation to health, well-being and resilience and it is thought that they may contribute to protecting against illness and promoting good quality of life. For example, meta-analysis indicates that healthy individuals show an attentional bias favoring positive over neutral information [3] and positive attentional bias improves resilience to stress [4]. Inducing a positive attention bias enhances optimism [5] and attending to happy faces is associated with greater life satisfaction [6]. Likewise, positive interpretation bias predicts well-being [7], healthy individuals make more positive than negative interpretations of social situations [8] and more positive interpretations are associated with lower levels of anxiety and paranoia [2]. Insufficient work has been conducted on the link between bias and well-being in Eastern samples to draw decisive conclusions, but those which have replicate the Western pattern of findings [9].

Negative (or attenuated positive) cognitive biases have been extensively studied in relation to psychopathology. Considerable evidence shows that anxious individuals preferentially allocate attention towards threat cues and interpret emotionally ambiguous information in a negative manner [10], [11], [12]. These negative cognitive biases are one of a variety of mechanisms proposed as contributing to the cause and maintenance of psychological disorders by supporting dysfunctional beliefs that elicit negative automatic thoughts. Although limited in quantity, those studies investigating bias and psychopathology in Eastern cultures, have replicated the pattern of findings in the West. For example, two studies have focused on anxiety in adolescence and childhood in Chinese culture. Both reported negative interpretations of ambiguity were associated with higher levels of childhood anxiety [13], [14].

In the present study we sought to first establish the broad profile of bias in healthy cross-cultural comparisons in order to establish an appropriate baseline against which to subsequently measure patterns associated with both well-being and pathology in Eastern versus Western cultures. The link between biases in the general population versus those associated with disorder and how these have been separately investigated has been considered in detail (in the context of attention) in an extensive review of both areas [12], together with recommendations for future research work in each area. Without a normative baseline one would not know whether cultural differences in psychopathology or well-being related bias were truly reflecting a culturally different profile or were simply a manifestation of broader cross-cultural differences. Establishing the broad profile of biases in a healthy sample therefore provides an essential platform from which to subsequently investigate associated effects. As such biases related to psychopathology and well-being served as an important motivator for the present investigation, but were not the topic of our investigation.

Another important context for the current investigation is cross cultural research conducted within the field of social psychology. This suggests that some aspects of human cognition are universal [15], [16], but there are also significant cultural differences. Western societies are individualistic, giving priority to the self over the group, whereas East Asian societies are collectivistic, prioritising the group [17]. These differences manifest in patterns of cognition and behavior concerning the self and others [18], [19]. East-West differences in the motivation to self-enhance–to maintain high self-esteem and a positive self-image–are also well established. A recent meta-analysis concluded that Westerners were strongly motivated (*d* = .87 for a self-serving bias) to view themselves positively across a wide range of measurement methods, but that East Asians showed no self-enhancement effects [20].

Cultural differences clearly have a bearing on many aspects of cognition, leading us to expect differences in attentional and interpretative biases too. The literature reviewed above suggests two alternative possibilities. On one hand, cultural differences in self-enhancement predict that Westerners will be more positively biased than East Asians. The motivation to see oneself in a positive light might manifest as making positive interpretations of ambiguous information and selecting positive information for attentional processing. Consistent with this, Westerners tend to be optimistic in the prediction of both negative and positive future events, while East Asians are pessimistic [21]. Likewise, East Asians attend more to avoidance-oriented information, whereas Westerners attend more to approach-oriented information [22].

On the other hand, lower prevalence rates of mental health disorders in East Asia than in the West [23], [24] suggest the reverse hypothesis; that East Asians would be the more positively biased group on experimental measures of interpretation and attention. If negative cognitive biases are considered a maintaining mechanism for psychological disorder, as explained and evidenced earlier, one would expect cultures with lower disorder prevalence to exhibit correspondingly healthier cognitive biases. Our study was designed to distinguish these hypotheses by measuring cognitive biases in East Asian and Western samples. In doing so we hoped to inform the cultural appropriateness of psychological interventions and well-being programs.

In an increasingly globalized world migration might play an important role in normalizing any cultural differences and we therefore also examined whether migration alters bias. In 2015, 3.3% of the world's population lived outside their country of origin [25]. Immigrant populations have a higher incidence rate of psychiatric disorders than local populations [26]. The cause of increased mental health problems in immigrant populations is unclear and varies from disorder to disorder but is likely to involve multiple factors. One suggested factor is the way in which a new culture can cause change in cognitions and behavior through acculturation. Acculturation refers to the process of cultural modification in which individuals who are part of a minority group adapt to the culture of the majority [27], [28]. This highlights the need both to understand other cultures, but also to understand the process of acculturation itself. Recent work has shown that modification of cognitive biases is possible, with corresponding consequences for well-being [29], [30]. This confirms that it is, in principle, possible to observe acculturation effects on cognitive biases, even in the short-term, and illustrates the importance of investigating the form they take.

The current study used two testing sites to compare local East Asian (Hong Kong) and Western (UK) samples on measures of attention and interpretation bias to see whether a difference exists. In addition, we assessed whether acculturation affects any cross-cultural differences by testing both short-term (< 2 months) and long-term (> 2 years) migrants in the respective sites. To measure each type of bias we used well-established experimental tests that have been specifically adapted and validated for a Chinese sample [31]. We addressed the following two questions:

- are there cultural differences between East Asians and Westerners in their pattern of biased cognition, either for attention or interpretation?
- is there evidence of acculturation of cognitive biases, either in East Asian or Western migrants?

## Methods

### Participants

Participants were recruited at each site (UK, Hong Kong) through institutional email circulars, targeted advertisement within relevant expatriate organizations, and posters at universities and local venues. Interested participants were invited take part if: they were aged over 18; spoke fluently in their native language; their scores on the State-Trait Anxiety Inventory (STAI) and Beck Depression Index (BDI) were below 60 (2 subsequently scored 60 at the time of testing and were replaced) and 30, respectively (to avoid sample contamination by pathologically negative biases); and had no previous head injuries; and no major physical or mental illness (past or current). Participants were recruited into one of 6 groups; local UK, local Hong Kong (HK), short-term HK migrants to the UK, long-term Hong Kong migrants to the UK, short-term UK migrants to HK and long-term UK migrants to HK. Criteria for group eligibility were as follows:

- Local samples: nationals who had not themselves resided outside their home country for more than 1 month and whose families had resided permanently in the home country for at least the last 2 generations.
- Short-term migrants: nationals of the home country (with families residing there for at least the last 2 generations) who had migrated to the host country within the last 2 months.
- Long-term migrants: nationals of the home country (with families residing there for at least the last 2 generations) currently living in the host country for a continuous period of at least 2 years.

We conducted an a priori power calculation to determine sample size, in line with funding requirements. Calculations were performed in G*Power 3 [32]. Cross-cultural differences in biased cognition were expected from a Group (UK, HK) x Direction (positive bias, negative bias) within-between interaction using a repeated measures ANOVA. We required 90% power to detect a medium effect, with alpha = 0.05, and an expected correlation between repeated measures of 0.7. With these parameters the required sample size was 28 participants per group, and we therefore aimed to recruit 30 per group. However because different analytical designs produce different levels of achieved power, and our actual final sample size was usually larger than 30, we also present sensitivity analyses within the results. These show the detectable effect size for each specific analysis, given the other parameters of the design.

### Materials

Full details of the task development including back translation procedures and validation for the Chinese word versions can be found in [31] and the materials themselves are available online in the Open Science Framework: https://osf.io/eca3t/.

#### Interpretation bias

The Similarity Rating Task (SRT) [33] comprised 15 emotionally ambiguous text passages designed to measure the degree of interpretation bias. The task has adequate reliability in both English and Chinese versions (0.56–0.66 split half) and bilingual participants’ performance in both languages was similar, demonstrating good measurement equivalence.

After reading three sentences of text setting up an ambiguous social situation participants completed a fragmented word, by filling in the first missing letter or Chinese character, and answered a yes/no comprehension question about the passage, thus ensuring the ambiguous meaning had been maximally encoded. For example:

*You give a presentation during class*. *People look interested and applaud at the end*. *However*, *you feel you cannot answer the last… qu-s-i-n* (question). *Did you give a presentation during class*? *Yes/No* (Correct response: yes.)

To assess the interpretation made of each passage participants rated how similar to the original were two differently disambiguated sentences (called targets) on a 4 point Likert scale (1 = very different to 4 = very similar). In the above example targets were: ‘*Your presentation is successful*’ (positive interpretation) and ‘*Your presentation is unsuccessful’* (negative interpretation). Two further sentences (called foils) controlled for response bias by assessing any general tendency to give differential endorsement of positive or negative material. In the above example, foils were: *‘You are generally a good writer’* (positive) and ‘*You are generally a bad writer’* (negative). A positive interpretation bias is inferred from higher ratings given to positive than negative targets.

The Scrambled Sentences Task (SST) [34]; involved reordering words (or Chinese characters) to make meaningful sentences with either a positive or negative meaning. Smith and colleagues [31] report adequate reliability of the English and Chinese versions (.58-.73 split half) and bilingual participants’ performance in both languages was similar, demonstrating good measurement equivalence.

Participants viewed 15 strings of mixed up words/characters and had to unscramble them to create meaningful sentences using only five out of the six, completing as many as possible in 15 minutes. Word strings were designed such that two different sentences were possible, with positive and negative meanings, respectively depending on the words used. For example, ‘*myself in disappointed am confident I’* could be unscrambled into ‘*I am confident in myself*’ (positive interpretation) or ‘*I am disappointed in myself*’ (negative interpretation). Participants performed the task under cognitive load (remembering a six-digit number) which is known to increase task sensitivity by preventing any tendency to suppress or control bias.

#### Attentional bias

Following the recommendation of Smith and colleagues [31] the current study used both word and picture (emotional faces) versions of each attentional task. Details of materials used for the picture versions of each task are given in Supporting information, ‘Method, Materials’.

The emotional Stroop task [35] measures attentional competition by comparing time taken to name the color of a series of emotional items with time taken to name the color of a series of neutral items. If greater attention is directed toward the content of emotional items, then this should produce greater impairment of color-naming performance on these, compared to neutral, items.

Materials for the word version of the task included four block arrays of 20 socially threatening (e.g. ‘disgusted’), 20 physically threatening (e.g. ‘cancer’), 20 positive (e.g.’ happiness’), and 20 neutral (e.g. ‘curtain’) words shown in blue, red, yellow or green on a white background. Each array was presented 4 times in total. Order of individual words within the array and ink color assignment were randomized separately in each block presentation. In both versions, participants had to name the color of each individual item in the block using their native language, as quickly as possible, reading from left to right across each row. Time taken to color-name each block was recorded using a stopwatch.

The Attentional Probe Task (APT) [36] measures reaction time to identify a probe letter appearing in the prior location of one or other of a pair of stimuli presented either side of a fixation cross. Time taken to identify the probe should be faster if attention is already allocated to that side of the visual field. For instance, faster reaction times for trials where the probe replaces threatening stimuli, in comparison to neutral stimuli, would indicate an attentional bias towards threat.

Word materials comprised 12 social threat (e.g. ‘gossip’), 12 physical threat (e.g. ‘fatal’), 12 positive (e.g. ‘loving’) and 12 neutral (e.g. ‘pepper’) words. See Supporting information ‘Materials’ for details of the picture version. In both versions of the task, a fixation cross (1000ms) was followed by pairs of emotional–neutral stimuli presented for 500ms on either side of fixation. A probe letter (E or F) then replaced the stimulus pair, appearing in either the previous emotional or neutral location. Participants identified the letter as quickly as possible, but without making mistakes and millisecond reaction times were recorded with an E-Prime response box.

#### Questionnaire measures

To estimate intelligence participants completed two language-independent subtests (block design and matrix reasoning) from the Wechsler Abbreviated Scale of Intelligence [37] to assess their Perceptual Reasoning Index as a proxy for IQ.

The State-Trait Anxiety Inventory–Trait version (STAI-T) [38] is a 20-item self-report scale used to measure trait anxiety. The C-STAI-T was used for Chinese-speaking participants [39]. Higher scores represent more severe trait anxiety. Internal consistency coefficients range from .86 to .95 and test-retest reliability coefficients range from .65 to .75 over a 2-month interval [38]. Internal consistency for the Trait scale in the C-STAI has been shown to be high (.83 [39]).

The Beck Depression Inventory [40] is a 21-item self-report rating inventory that measures symptoms of depression. Chinese-speaking participants completed the Chinese version of the BDI [41]. Higher scores represent more severe depression. The BDI demonstrates high internal consistency (.81) for non-psychiatric populations [40], and the Chinese version also has high internal consistency (.77) and good reliability (.86 [41]).

### Procedure

Ethical approval was granted by King’s College London (KCL) Research Ethics Committee (Ref: PNM/13/14-74), and the research was conducted in accordance with the Declaration of Helsinki. After signing written informed consent, participants undertook the six cognitive tasks in counterbalanced order, followed by the questionnaires. Participants completed all cognitive tasks on a Fujitsu laptop running E-Prime 2.0 [42] with the exception of the SST which was delivered as a pen-and-paper test. Average session length was 2 hours, and sessions were conducted in the participant’s native language. Participants were compensated at a flat rate of £20 (or Chinese equivalent).

## Results

### Question 1: Are there cultural differences in biased cognition?

Seventy-five eligible local participants completed the study, comprising 36 UK participants and 39 HK participants (Table 1). HK participants reported significantly higher trait anxiety than the UK sample therefore STAI-T was used as a covariate Higher levels of self-report anxiety in East Asians is a well-documented [43] but currently unexplained [44] phenomenon. It is not clear whether the difference reflects genuine differences in levels of distress/ impairment or is a consequence of social construction or response bias. As a result it is difficult to know whether or not to use this variable as a covariate. Further, Miller and Chapman [45] have argued that any variable systematically related to a grouping variable should cannot be used as a covariate without corrupting the grouping variable itself (since this would remove variance legitimately associated with the grouping variable). We therefore reran our main analyses without any covariates. This revealed a largely similar pattern of data, a summary of which is provided in the supplementary material, S1 File. Where relevant, data met the assumption of homogeneity of variance.

**Table 1:** Socio-demographic characteristics and individual difference measures for the sample. Mean (standard errors).

							Hong Kong Migrants to UK^1^					UK migrants to Hong Kong^1^				
Group Characteristic		UK (n = 36)	95% CI	Hong Kong (n = 39)	95% CI	*p*	ST (n = 37)	95% CI	LT (n = 31)	95% CI	*p*	ST (n = 31)	95% CI	LT (n = 28)	95% CI	*p*
Age		23.39 (5.23)	[21.62, 25.16]	25.64 (11.22)	[22, 29.28]	>.250	25.41 (4.00)	[24.07, 26.74]	27.29 (3.97)	[25.83, 28.75]	0.157	22.55 (5.32)	[20.60, 24.50]	36.71 (9.04)	[33.21, 40.22]	**< .001**
Male (female)		9 (27)		16 (23)		0.145	8 (29)		7 (24)		0.200	13 (18)		17 (11)		**0.040**
Education																
	GCSE	1		1			0		0			0		0		
	A Levels	0		2			0		0			0		0		
	Higher Education	35		36		0.089	37		31		**< .001**	31		28		**< .001**
Employed (unemployed, inc students)		20 (16)		13 (26)		>.250	22 (15)		26 (5)		**< .001**	8 (23)		27 (1)		**< .001**
IQ		112.25 (10.23)	[108.79, 115.71]	108.21 (14.68)	[103.45, 112.96]	0.174	113.14 (9.58)	[109.94, 116.33]	116.68 (9.16)	[113.32, 120.04]	**0.021**	109.23 (14.23)	[104.01, 114.44]	110.68 (11.37)	[106.27, 115.09]	p>.250
STAI-T		35.64 (9.08)	[32.57, 38.71]	44.18 (6.80)	[41.98, 46.38]	**< .001**	43.57 (8.28)	[40.81, 46.33]	45.81 (7.84)	[42.93, 48.68]	**< .001**	43.03 (99.35)	[39.60, 46.46]	38.50 (8.42)	[35.24, 41.76]	**p < .001**
BDI-II		5.47 (6.03)	[3.43, 7.51]	7.38 (5.72)	[5.53, 9.24]	0.163	7.43 (6.74)	[5.19, 9.68]	8.90 (5.13)	[7.02, 10.79]	0.136	8.94 (6.30)	[6.62, 11.25]	5.89 (5.38)	[3.81, 7.98]	p>.250

*Note*. CI = confidence interval. ST = Short-term migrant. LT = Long-term migrant. GCSE = General Certificate of Secondary Education; STAI-T = State-Trait Anxiety Inventory-Trait. BDI-II = Beck Depression Inventory. SESPS = Self-Enhancement and Self-Protection Strategies Scale.

^*1*^ *p* values reflect one way ANOVAs comparing the two local and two migrant samples, in accordance with the 4 group design of the acculturation analyses. Variables differing significantly across the four relevant groups were used as covariates in acculturation analyses.

### Achieved power and sensitivity

Post hoc power analysis showed that for a sample size of 75 (SST) we achieved 99% power to detect a medium effect size on a within-between interaction at alpha = 0.05 assuming a repeated measurements correlation of 0.7. Dropping the sample size to 73 (SRT) made no detectable difference to achieved power. At 90% power, assuming the same other parameters, our analyses were sensitive to detect small (f = 0.1) to medium (f = 0.25) effects (detectable fs = 0.15 for SRT, 0.14 for SST, 0.13 for emotional Stroop and attentional probe).

#### Interpretation bias

On the Similarity Rating Task (SRT) two participants’ data were missing due to equipment failure. Errors and outliers are not possible by virtue of task design. Mean similarity ratings were automatically calculated per participant per condition (Target Type: target, foil x Disambiguation Direction: positive, negative) using E-Prime v20 Data Aid. A target bias score, reflecting interpretation of ambiguity, and a foil bias score, reflecting response bias, were calculated by subtracting negative from positive sentence disambiguation ratings [46]. A larger bias score reflects a stronger positive bias. A Group (UK, HK) x Bias Type (target bias, foil bias) repeated measures ANCOVA showed no evidence of cultural differences on this task. Non-significant effects were as follows. Group main effect, *F*(1, 70) = 1.89, *p* = .173, η_*p*_^*2*^ = .026; Group x Bias Type interaction, *F*(1, 70) = 0.07, *p* = .797, η_*p*_^*2*^ = .001. Means and standard deviations are provided in Table 2.

**Table 2:** Means (standard deviations) on similarity ratings task (similarity ratings, 1, different– 4, similar).

	UK (n = 35)	95% CI	Hong Kong (n = 38)	95% CI	Hong Kong migrants to UK				UK migrants to China			
					ST (n = 37)	95% CI	LT (n = 31)	95% CI	ST (n = 31)	95% CI	LT (n = 28)	95% CI
Target Bias	0.53 (0.49)	[.28, .63]	0.50 (0.49)	[.41, .73]	0.46 (0.41)	[.33, .63]	0.37 (0.43)	[.19, .54]	0.40 (0.37)	[.33, .62]	0.55 (0.37)	[.16, .51]
Foil Bias	0.44 (0.24)	[.29, .52]	0.52 (0.38)	[.44, .66]	0.57 (0.37)	[.47, .69]	0.53 (0.32)	[.39, .64]	0.48 (0.30)	[.46, .67]	0.47 (0.34)	[.36, .61]

*Notes*: CI = confidence interval. ST = Short-term. LT = Long-term

On the Scrambled Sentences Task (SST) task design precludes outliers and errors are incorporated in the bias score (i.e. counted under ‘total attempted’). Positivity bias and negativity bias scores were calculated [47], [46] by calculating the proportion of sentences unscrambled to create a positive or negative meaning out of the total attempted, with higher values indicating stronger bias. We used the inclusive scoring method in which both exact matches, and matches with similar meaning, were included in this count (e.g. ‘someone was aggressive toward me’ or ‘someone was aggressive’ would both be counted as negative interpretations). Note that the positivity bias scores and negativity bias scores are not simply the inverse of each other, because some responses are counted as errors (neither positive nor negative interpretations) and are therefore included only in the denominator.

A Group (UK, HK) x Bias Type (positivity bias, negativity bias) ANCOVA revealed a significant interaction, *F*(1, 72) = 5.15, *p* = .026, η_*p*_^*2*^ = .07. Simple main effects of Bias Type showed that groups differed on positivity bias scores, *F*(1, 72) = 8.14, *p* = .006, η_*p*_*^2^* = .10, but not negativity bias scores *F*(1, 72) = 2.10, *p* = .152, η_*p*_*^2^* = .03. HK were significantly more positively biased (75% sentences unscrambled positively) than UK (68% sentences unscrambled positively). Means and standard deviations are provided in Table 3.

**Table 3:** Means (standard deviations) on scrambled sentences task (proportion of sentences).

	UK (n = 36)	95% CI	Hong Kong (n = 39)	95% CI	Hong Kong migrants to UK				UK migrants to China			
					ST (n = 37)	95% CI	LT (n = 31)	95% CI	ST (n = 31)	95% CI	LT (n = 28)	95% CI
Positivity Bias	0.68 (0.16)	[.58, .71]	0.75 (0.20)	[.72, .84]	0.62 (0.19)	[.57, 69]	0.61 (0.19)	[.55, .69]	0.72 (0.03)	[.66, .79]	0.74 (0.04)	[.66, .81]
Negativity Bias	0.19 (0.17)	[.17, .28]	0.21 (0.18)	[.11, .22]	0.12 (0.13)	[.07, .16]	0.13 (0.12)	[.06, .17]	0.18 (0.03)	[.12, .24]	0.21 (0.04)	[.14, .28]

*Notes*: CI = confidence interval. ST = Short-term. LT = Long-term

#### Attentional bias

Details of analyses, results and tables of means for the picture versions of each attentional task are given in supporting information, S1 Table. There were no significant results.

On the emotional Stroop task median reaction times (RT) were used to create a bias score for each emotion type by subtracting the neutral from the emotional RT (Table 4). A larger bias score reflected greater attentional bias favoring the corresponding emotion. ANCOVA revealed a significant Bias Type (social threat, physical threat, positive) x Group interaction, *F*(2,144) = 4.17, *p* = .017, η_*p*_*^2^* = .055. Pairwise comparisons showed no significant differences between groups on individual emotion types (*p*s > .07). The interaction was therefore interpreted by considering simple main effects of Group examined as linear trends across bias types. These were significant in both the UK, *F*(1, 35) = 4.19, *p* = .048, η_*p*_*^2^* = .107, and the HK samples, *F*(1, 38) = 6.84, *p* = .013, η_*p*_*^2^* = .152, with opposite gradients. The UK sample showed greatest interference for social threat, waning to reduced interference for positive material. HK showed the reverse pattern, with strongest interference for positive material and weaker interference for threat (Table 4).

**Table 4:** Means (standard deviation) on emotional stroop task (median emotional interference scores, seconds).

	UK		Hong Kong		Hong Kong migrants to UK				UK migrants to Hong Kong			
	(n = 36)	95% CI	(n = 39)	95% CI	ST (n = 37)	95% CI	LT (n = 31)	95% CI	ST (n = 31)	95% CI	LT (n = 28)	95% CI
Positive	-0.26 (0.68)	[-.57, .06]	0.05 (1.02)	[-.25, .35]	-.00 (0.97)	[-.32, .28]	0.06 (0.98)	[-.36, .36]	-0.11 (1.21)	[-.55, .33]	0.23 (0.77)	[-.07, .53]
Social Threat	0.06 (0.84)	[-.26, .36]	-.30 (0.89)	[-.58, .01]	-.04 (0.93)	[-.36, .29]	-.00 (1.32)	[-.25, .51]	-0.15 (1.12)	[-.58, .10]	0.52 (0.73)	[.23, 1.08]
Physical Threat	0.01 (0.84)	[-.35, .38]	-.23 (1.16)	[-.58, .12]	0.14 (0.83)	[-.21, .49]	.01 (1.34)	[-.42, .40]	-0.04 (1.07)	[-.42. .39]	0.70 (1.09)	[.22, 1.22]

*Notes*: CI = confidence interval. ST = Short-term. LT = Long-term. UK = United Kingdom. CI = Confidence Interval

On the Attentional Probe Task data cleaning (see Supporting information, ‘Attentional probe data cleaning’) applied the same principles for UK and HK samples, after which the data were suitable for parametric analyses. Emotion bias scores, for each emotion type, were calculated by subtracting RTs when the emotion location was probed from those when the neutral location was probed (Table 5). Larger bias scores therefore reflected selective attending to the emotional material. A Group (UK, HK) x Emotion (positive, social threat, physical threat) ANCOVA showed a significant interaction, *F* (2,144) = 3.72, *p* = .027, η_*p*_*^2^* = .049. Simple main effects of Emotion showed that groups did not differ on biased attention to physical or social threat, all *ps* > .15, but the HK sample showed significantly stronger positive attentional bias than their UK counterparts, *t* (73) = -2.38, *p* = .020, Cohen’s *d* = 0.56.

**Table 5:** Means (standard deviation) on attentional probe task (reaction time difference score: Mean neutral minus mean emotional trial, msec).

	UK		Hong Kong		Hong Kong migrants to UK				UK migrants to Hong Kong			
	(n = 36)	95% CI	(n = 39)	95% CI	ST (n = 37)	95% CI	LT (n = 31)	95% CI	ST (n = 31)	95% CI	LT (n = 28)	95% CI
Positive	-5.74 (22.68)	[-14.97, 1.42]	6.86 (23.09)	[-.014, 15.66]	1.73 (31.46)	[-7.58, 10.63]	-1.39 (33.10)	[-13.81, 7.73]	-1.78 (21.44)	[-11.33, 7.24]	-2.65 (31.66)	[-18.08, 5.14]
Social Threat	-10.81 (17.68)	[-19.30, 2.90]	-5.25 (26.71)	[-12.83, 2.85]	-5.80 (25.65)	[-13.72, 2.28]	2.15 (25.91)	[-6.41, 12.51]	-9.52 (25.72)	[-20.01, 1.87]	-1.96 (25.70)	[-10.19, 12.50]
Physical Threat	5.40 (26.86)	[-3.60, 15.08]	-3.35 (25.18)	[-12.60, 5.26]	-12.69 (30.74)	[-20.98, -3.19]	-2.08 (26.72)	[-8.51, 12.53]	-5.74 (18.42)	[-16.59, 2.25]	2.91 (27.53)	[-8.24, 15.33]

*Notes*: CI = confidence interval. ST = Short-term. LT = Long-term. UK = United Kingdom. CI = Confidence Interval

### Question 2: Is there evidence of acculturation?

**Fig 1:**
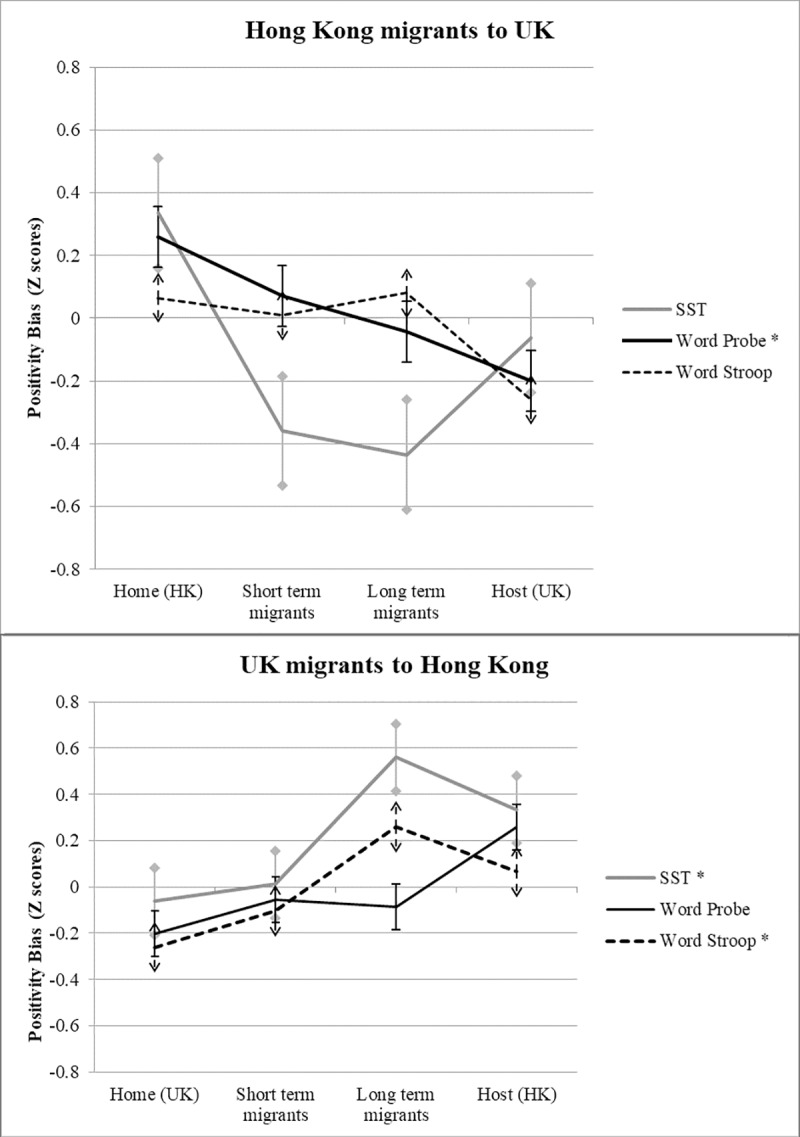
Pattern of acculturation effects across three measures of cognitive bias. (A) Hong Kong migrants to UK. (B) UK migrants to Hong Kong. Values are standardized values (Z scores) of the bias score means provided in Table 2, to allow meaningful comparison of the pattern of data across tasks with different dependent measures. Abbreviations: SST–scrambled sentences task. HK–Hong Kong. UK–United Kingdom. * indicates this task showed a significant linear trend at *p* < .05.

In assessing acculturation, we considered only those tasks in which cross cultural differences were observed. Where cognitive biases were already similar across cultures, there would be no basis to suggest that they would be altered by migration to the opposite culture (nevertheless we tested the other dependent variables in the same manner and none showed significant effects of acculturation). We considered each culture separately and added the two relevant short and long-term migrant groups into the preceding local comparison analyses, permitting a direct comparison of relevant migrant groups with both their host and native cultures. Univariate ANCOVAs were conducted on relevant bias scores (SST positivity bias, Word Stroop positive bias, word attentional probe positive bias) to compare Group (UK, HK, relevant short-term migrants, relevant long-term migrants) performance and including as covariates any demographic variables that also differed significantly across groups (see Table 1) . Significant main effects of Group were investigated using Bonferroni adjusted pairwise comparisons. We also tested the hypothesis that acculturation effects would be reflected by a systematic pattern in which bias scores shifted away from the home culture and towards the host culture, by ranking the groups in the order native, short-term migrant, long-term migrant and host and conducting linear trend analyses. Results are shown in Fig 1A and 1B using standardized scores. Finally, in a post-hoc confirmation check of our findings (see supporting information, S1 Fig) we conducted regression analyses, for each migrant sample separately, to test whether duration of migration predicted size of positive bias.

#### Achieved power

Post hoc power analysis for HK migrants to the UK showed that we had power to detect a medium effect size at alpha = 0.05 as follows: univariate ANOVA 70%; linear trend 98%, regression (see Supporting information) 88%. The equivalent values for the UK to HK migrant sample were 67%, 97% and 83%.

#### HK migrants to the UK

Sixty eight East Asian migrants completed the study, 31 long-term and 37 short-term. By design, long-term migrants had resided significantly longer in the UK (median 60 months, range 25 months -16 years), than short-term migrants (median 30 days, range 9–60 days).

#### Interpretation bias

For the Scrambled Sentences Task (SST) the Group main effect was significant, *F*(3, 136) = 5.37, *p* = .002, η_*p*_*^2^* = .11. Follow-up comparisons revealed that neither short- nor long-term migrants differed significantly from the UK sample, *ps* > 0.999, but both migrant groups were significantly less positively biased than the HK sample. Short-term East Asian migrants created 14% fewer positive sentences than their home HK counterparts, *p* = .005 [95% CI -.256, -.031] and long-term migrants created 15% fewer positive sentences than their local counterparts, *p* = .010, [95% CI -.278, -.025]. Thus both migrant groups appeared to have acculturated to the UK cultural norm, showing a reduced native positive bias which now matched that of the local population. The linear trend was not significant, *p* = .090, consistent with the pattern described above in which both migrant groups were already performing similarly to the UK sample.

#### Attentional bias

For the word emotional Stroop task a univariate ANCOVA showed no significant Group differences, *F*(3, 136) = 0.23, *p* = .89, η_*p*_*^2^* = .005, and no linear trend on contrast tests, *ps* > .140. There was therefore no evidence of acculturation effects on emotional Stroop interference arising from positive emotional material.

For the word Attentional Probe Task there was no significant Group main effect, *F*(3, 136) = 1.03, *p* = .380, η_*p*_^2^ = .022. There was a significant linear trend, *t*(129.53) = 2.15, *p* = .034, suggesting that a longer migration period was associated with a bias more like that of the UK host culture.

#### UK migrants to Hong Kong

Fifty nine UK migrants completed the study, 28 long-term and 31 short-term. By design, long-term migrants had resided significantly longer in East Asia (median 42 months, range 24 months-33 years) than short-term migrants (median 53 days, range 30–75 days). The approach to analysis was as above, but using the Group factor levels: UK, Hong Kong, short-term migrants to HK, long-term migrants to HK. Results are shown in Fig 1B.

#### Interpretation bias

For the Scrambled Sentences Task (SST) there was a significant main effect of Group, *F*(3, 125) = 3.37, *p* = .021, η_*p*_*^2^* = .08. Follow up comparisons revealed that home UK and Hong Kong samples differed significantly (*p* = .002) and there was a significant linear trend on contrast tests, *t*(88.18) = 3.18, *p* = .002, suggesting that a longer migration period increased the positivity bias of the UK migrants towards that of the Hong Kong host culture.

#### Attentional bias

For the word emotional Stroop task there was no significant Group main effect, *F*(3, 125) = 0.55, *p* = .65, η_*p*_*^2^* = .013, but a significant linear trend on contrast tests, *t*(100.99) = 2.50, *p* = .014, suggesting acculturation effects for UK migrants, whose positivity bias increased towards that of their Hong Kong hosts.

For the word Attentional Probe Task a univariate ANCOVA on positive bias scores showed no significant differences between levels of the factor Group, *F*(3, 125) = 1.22, *p* = .304, η_*p*_*^2^* = .03, and no linear trend on contrast tests, *p*s> .283.

#### Does migration duration predict strength of positive bias?

Please see supporting information S1 Fig and S1 File for full details of data preparation and results for this analysis. For HK migrants to the UK the duration of migration significantly predicted a combined index of positive bias, *F*(1, 29) = 76.78, *p* < .001, explaining 72% of the variance in bias (R^2^ = 0.73), *β* = 0.85, *t* = 14.03, *p* < .001. For UK migrants to HK the duration of migration also significantly predicted positive bias, *F*(1, 34) = 83.00, *p* < .001, explaining 71% of the variance in bias (R^2^ = 0.71), *β* = 0.84, *t* = 13.17, *p* < .001.

## Discussion

In this two-site cross-national study, we investigated cultural differences between East Asians and Westerners in their patterns of biased attention and interpretation. We also asked whether migrants to the other culture showed any evidence of acculturation of their cognitive biases. Broadly speaking, results suggested that individuals from Hong Kong were more positively biased than people from the UK in both attention and interpretation. For several tasks, and in both cultures, migrants’ positive biases were either more similar to their hosts than their home culture or showed a significant gradient in this direction; East Asian migrants had reduced positivity bias, while Western migrants had greater positivity when resident in the other culture. Within both migrant samples the duration of migration significantly predicted a combined index of positive bias.

More specifically, on one of two interpretation tasks (creating sentences from ambiguous word strings) people from Hong Kong were more positively biased than UK participants, unscrambling 75% (versus 68% for UK) of sentences into a positive meaning. When the task involved ambiguous passages we found no cultural differences. In a similarly mixed set of findings, two of four attentional tasks (those using word stimuli, but not those using faces) also revealed a cultural difference in positivity. On the word emotional Stroop, positive information was more salient than negative in Hong Kong, but in the UK we found the opposite effect. On the word Attentional Probe, East Asians showed a stronger positive bias (i.e. selecting positive over neutral information) than their Western counterparts.

These data are in line with the second of our two possible hypotheses; that East Asians would be more positively biased than Westerners, consistent with cultural differences in prevalence of psychological disorders. East Asian cultures are reported to have lower prevalence rates of mental health disorders compared to the West [23], [24]. According to many cognitive theories of psychological disorder, negative biases are considered a maintaining mechanism for psychological disorder and therefore cultures with lower prevalence of psychopathology would be predicted to exhibit a correspondingly less negative/ more positive profile of biased cognition. Our results are consistent with this and with assumptions that the close coupling between biased cognitive processing of emotion and mental well-being, as observed in the West, is a universal phenomenon. Theories of cognitive bias predict that positive East Asian biases would be helping to maintain the population’s lower levels of psychopathology and this possibility warrants further direct investigation.

In contrast, our data did not follow the pattern expected from the self-enhancement literature, and there are a number of possible reasons for this. Firstly, the materials used may not have been relevant to the self-enhancement motive. For example, it is not obvious how self-esteem would be boosted by attending to neutral objects (e.g. flowers, books) rather than threat (e.g. snake, gun) during the attentional probe task. While the sentences used in the interpretation task were more explicitly self-referent, participants performed this task under cognitive load, precisely to prevent contamination from the kind of strategic processing which is most likely involved in self-enhancement. Secondly, the tasks may measure more implicit processes than those involved in self-enhancement. Indeed, Heine and Hammamura’s [20] meta-analysis found that cross-cultural differences did not emerge on self-enhancement measures involving *implicit* self-esteem.

Possible acculturation effects were found in attention and interpretation in migrant groups to both cultures. Specifically, East Asian migrants to the UK created over 10% fewer positive sentences than their non-migrant counterparts. This effect appeared quickly, in that both short- and long-term migrant groups showed a reduced positive bias, which was indistinguishable from the UK cultural norm. Differences on the word attentional probe task showed an incremental pattern in which long term migrants performed most like their UK hosts, showing greatest attention toward negativity. UK migrants to Hong Kong showed the opposite gradient of group differences, again consistent with acculturation, on one attention (word emotional Stroop) and one interpretation (Scrambled sentences) task. Supporting these conclusions, our post-hoc analysis of migrant samples alone showed that the length of the migration period significantly predicted a composite index of positivity bias, accounting for upwards of 70% of the variance in positivity.

Our study had a number of limitations. First, while the study was sufficiently powered to detect the required interaction effects for cross cultural differences, the sample size was nevertheless small. Further work is needed both to replicate our current main findings and address the distinct but related question of biases associated with psychopathology in different cultures. Although much larger sample sizes will be needed the current work should provide useful variance estimates upon which to base future sample size calculations. We also used STAI-T as a covariate in our analyses because we did not want to misattribute group differences in bias to cross cultural effects, if they were a consequence of co-occurring group differences in anxiety. However, this is controversial. Miller and Chapman [45] have argued that any variable systematically related to a grouping variable cannot be used as a covariate without corrupting the grouping variable itself (since this would remove variance legitimately associated with the grouping variable). Re-running the main analyses without covariates revealed largely the same pattern of results, nevertheless we acknowledge this issue as a limitation of our study. Likewise the ubiquitous finding that East Asians generally do self-report higher levels of anxiety than Westerners [43] requires an explanation that only future research will be able to provide [44] and this also limits the interpretation of the present findings.

In our acculturation analyses power was particularly low for our four-way between group comparisons, however we were able to supplement these analyses with better powered tests using linear contrasts and regressions. Furthermore, we only sampled one example of each culture. Hong Kong was colonized by Britain and is arguably a relatively ‘Western’ example of an East Asian culture. Although the UK still scores significantly higher on typically Western values than Hong Kong (e.g. individualism: 89 vs. 25) [48], our results need replication using different cultural examples of East Asians versus Westerners. Acculturation results relied upon cross sectional data and self-selection could have contributed to these effects. For example, migrants choosing to live in a different culture may already have characteristics matching that culture. Demographic differences in our migrant samples probably reflected their inherently different population profiles and results relied upon statistical correction. Most importantly, longitudinal studies are essential to evidence acculturation changes in a single sample of migrants followed up over time.

Also worthy of comment, results were present on some, but not all, of our bias measures. Cultural differences were absent on the text-based measure of interpretation and on picture (face) versions of the attention tasks. Acculturation effects were seen on only some of the tasks that differed across cultures. There are several possible reasons for these inconsistencies. Face processing is known to be a specialized process with unique characteristics [49]. Although facial expression recognition is consistent across cultures, there could be other substantial differences in attentional processing of faces, not necessarily applying to verbal processing. Differences across verbal tasks could be attributable to the near impossibility of obtaining ‘process-pure’ experimental measures [50], meaning that each task will tap a combination of different mechanisms, which could contaminate results. Alternatively, data could reflect genuine differences in the level at which cross-cultural effects operate. A further possibility is that effect sizes vary according to the task in use, meaning that despite our power calculations not all tasks achieved sufficient power for effects to be observed. Further research will need to distinguish these possibilities. Another approach is to combine measures of bias into a single bias index using standardized scores (as we did in our supplementary analyses, see S1 File), which arguably provides a more valid measure of the underlying construct, but loses the specificity afforded by measuring each bias individually.

Further work is needed to replicate the present findings with larger sample sizes and sampling a wider range of East Asian and Western populations. It will be important to conduct such work because if the pattern of results reported here proves robust, this raises the possibility that psychological well-being in Eastern cultures might be better protected than in the West. Our results also raise the possibility that the increasing internationalization of society brings with it some important consequences for health and well-being. The cognitive biases one holds may depend not only on one’s own cultural background, but also upon the culture within which one resides. Westerners may reap some degree of cognitive benefit by residing in East Asian cultures, while East Asian migrants may experience some potentially detrimental effects. A natural extension of the current work, once replication has confirmed these findings, would be to examine whether recent innovations involving modification of cognitive biases [51], [52] could be adapted to protect East Asian migrants to the West and improve resilience in Westerners.

## Supporting information

S1 File Supplementary methods and results. (DOCX)

S1 Fig Relationship between duration of migration and composite measure of positive bias. (DOCX)

S1 Table Means (standard deviation) on picture attentional bias tasks. (DOCX)
