# Supplementary material for: Biased cognition in East Asian and Western cultures
Source: PLoS One. 2019 Oct 15;14(10):e0223358. doi: 10.1371/journal.pone.0223358 (PMC6793946; doi:10.1371/journal.pone.0223358)
Supplement: S1 File — (DOCX) [file pone.0223358.s001.docx]

**S1 File Supporting information**

# Method

## **Materials**

**Picture version of emotional Stroop task.** Materials for the picture version of the task were taken from NimStim (Tottenham *et al.*, 2009) for UK participants and from the Japanese and Caucasian Facial Expressions of Emotion (JACFEE) and Japanese and Caucasian Neutral Faces (JACNeuF) series (Matsumoto & Ekman, 1988) for Chinese participants. Stimuli comprised four block arrays of 20 angry, 20 fearful, 20 sad, 20 happy and 20 neutral faces. Each face was given a color wash tint (blue, red, yellow or green) and appeared in a random location within each block presentation.

**Picture version of attentional probe task (APT).** Picture materials comprised four male and four female faces taken from the Montreal Set of Facial Displays of Emotion (Beaupré & Hess, 2005), each displaying facial expressions of happiness, sadness, fear, anger and neutral. Different emotional stimuli sets were used in each task in order to avoid repetition of identical stimuli, which is known to lead to response habituation. Separate culture-relevant sets were used for East Asians and Westerners.

# Results

# Question 1

**Picture emotional Stroop task analysis.** A Group x Emotion (happy, sad, fear, anger) analysis showed no significant Group effect, *F*(1, 72) = .014, *p* = .907, *η_p_²* < .001, nor interaction, *F*(3, 216) = 2.37, *p* = .072, *η_p_²* = .032, suggesting no evidence of cultural differences on this task. As for the other analyses achieved power for the n=75 sample was 99% to detect a medium effect size on a within-between interaction at alpha = 0.05 assuming a repeated measurements correlation of 0.7. At 90% power and the same other parameters, this analysis was sensitive to detect small (f = 0.1) to medium (f = 0.25) effects; detectable f = 0.12.

**Attentional probe data cleaning.** Trials with probe identification errors and anticipatory (<200ms) or delayed reaction times ( >2.5 SDs above sample mean) were removed. UK word probe data removed were as follows: 4.3% (2.7% errors; 1.6% outliers, 2.5 SD threshold = 723ms). Chinese word probe data removed: 5.2% removed (2.6% errors; 0.1% outliers, 2.5 SD threshold = 726ms). UK picture probe data: 3.9% removed (2.1% errors; 1.8% outliers, 2.5 SD threshold = 742ms). Chinese picture probe data: 4.9% removed (2.4% errors; 2.5% outliers, 2.5 SD threshold = 726ms).

**Picture attentional probe task** **analysis**. The analysis showed no significant effects or interactions, all ps > .60 suggesting no evidence of cultural differences on this task. The achieved power and sensitivity for this analysis was the same as reported above, for the picture Stroop task.

**Summary of main analyses without using any covariates**.

Interpretation bias

Similarity Rating Task analysis – no change (results non-significant)

Scrambled Sentences Task analysis –weaker results. Difference between groups on positivity bias scores reduced to trend level (p=.081). Group x Bias Type interaction did not reach significance.

Attention bias

Picture tasks - no change (results non-significant)

Word Stroop – no change to results. Bias type x Group interaction gained more significance (p=.004). Same results obtained on follow up linear trends.

Word probe – no change to results. Group x Emotion interaction remained significant (p=.023), with follow up tests showing groups differed only on positive bias (HK sample significantly more positively biased than UK, p=.02)

# Question 2

**East Asian migrants to the UK.**

## **Covariates.** Groups differed significantly on IQ, anxiety and employment status (see Table 1), which were entered as covariates in subsequent analyses.

**UK migrants to Hong Kong.**

## **Covariates.** Groups differed significantly in age^[[1]](#footnote-1)^, level of anxiety, gender balance, level of education and employment status (largely due to UK long-term migrants to China being older, highly educated professionals) which were entered as covariates in subsequent analyses.

**Does migration duration predict strength of positive bias?** We calculated a single index of positive bias by standardizing and summing positive bias scores on the three tasks involved in the acculturation analyses (SST positivity bias, word Stroop positive bias, word attentional probe positive bias). The composite measure of positive bias was approximately normally distributed. Length of migration had a significantly skewed distribution due to our short and long term migrant selection criteria (see Figure S1). Around half the data (short term durations were all less than 3 months) was clustered at floor, and long term durations were spread over a long positive tail. We therefore log transformed this variable, resulting in an approximately normal distribution, suitable for analysis. A linear regression analysis was performed for each migrant group separately (n = 68 for HK migrants to UK; n = 59 for UK migrants to HK) with composite positive bias score as the dependent variable and log transformed duration (in months) as the predictor. As reported in the ms, for both migrant groups duration of migration significantly predicted composite positive bias: HK migrants to UK: *F*(1, 29) = 76.78, *p* < .001, R^2^ = 0.73, *β* = 0.85, *t* = 14.03 and UK migrants to HK: *F*(1, 34) = 83.00, *p* < .001, R^2^ = 0.71, *β* = 0.84, *t* = 13.17, *p*<.001. Figure S1 shows the scatter plots of the untransformed duration of migration against composite positive bias score for each migrant group.

# References

1. Beaupré, M. G., & Hess, U. (2005). Cross-cultural emotion recognition among Canadian ethnic groups. *Journal of Cross-cultural Psychology*, *36*, 355-370. doi:10.1177/0022022104273656.
2. Matsumoto, D., & Ekman, P. (Producer). (1988). Japanese and Caucasian Facial Expressions of Emotion (JACFEE).
3. Tottenham, N., Tanaka, J. W., Leon, A. C., McCarry, T., Nurse, M., Hare, T. A., . . . Nelson, C. (2009). The NimStim set of facial expressions: Judgements from untrained research participants. *Psychiatry Research*, *168*, 242-249. doi:10.1016/j.psychres.2008.05.006.

1. Since controlling for age may also control for time spent in Hong Kong, in doing this we may have inadvertently removed some of the effects of acculturation. We therefore reran the analyses in this section of the manuscript (UK migrants to HK) without controlling for age. There were no substantive differences in the results or patterns of significance. [↑](#footnote-ref-1)
