# Supplementary material for: Biased cognition in East Asian and Western cultures
Source: PLoS One. 2019 Oct 15;14(10):e0223358. doi: 10.1371/journal.pone.0223358 (PMC6793946; doi:10.1371/journal.pone.0223358)
Supplement: S1 Fig — (DOCX) [file pone.0223358.s002.docx]

**Figure S1. Relationship between duration of migration and composite measure of positive bias.**


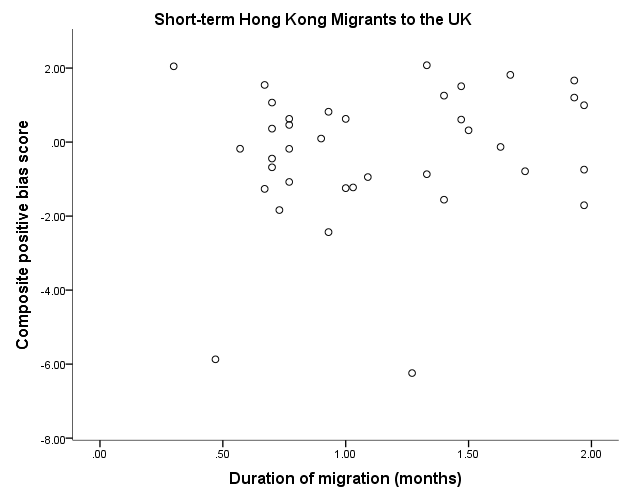


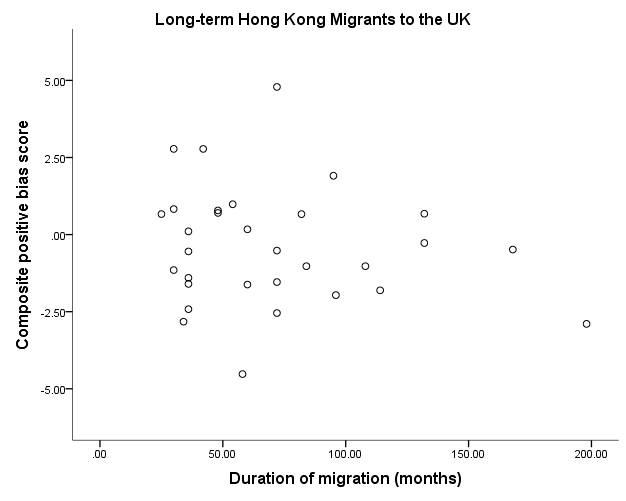


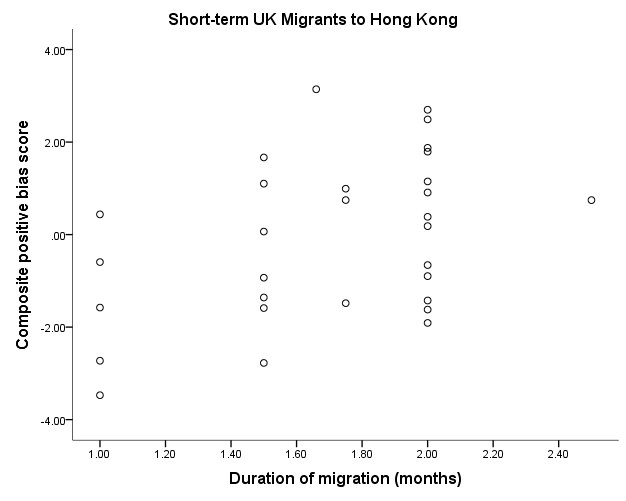


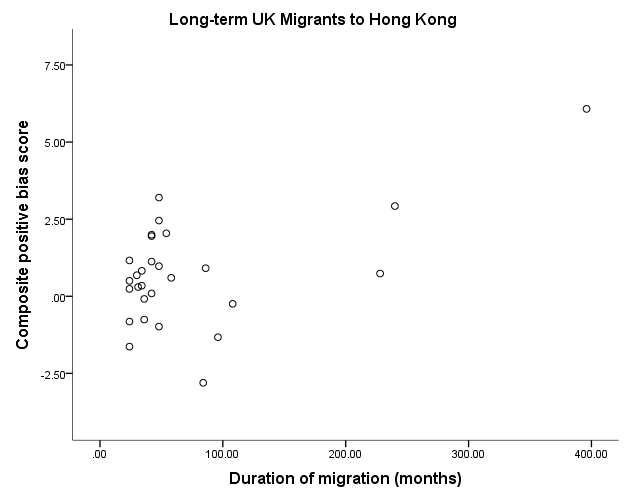


# References

1. Beaupré, M. G., & Hess, U. (2005). Cross-cultural emotion recognition among Canadian ethnic groups. *Journal of Cross-cultural Psychology*, *36*, 355-370. doi:10.1177/0022022104273656.
2. Matsumoto, D., & Ekman, P. (Producer). (1988). Japanese and Caucasian Facial Expressions of Emotion (JACFEE).
3. Tottenham, N., Tanaka, J. W., Leon, A. C., McCarry, T., Nurse, M., Hare, T. A., . . . Nelson, C. (2009). The NimStim set of facial expressions: Judgements from untrained research participants. *Psychiatry Research*, *168*, 242-249. doi:10.1016/j.psychres.2008.05.006.
