# Supplementary material for: Biased cognition in East Asian and Western cultures
Source: PLoS One. 2019 Oct 15;14(10):e0223358. doi: 10.1371/journal.pone.0223358 (PMC6793946; doi:10.1371/journal.pone.0223358)
Supplement: S1 Table — (DOCX) [file pone.0223358.s003.docx]

**Table S1. Means (standard deviation) on picture attentional bias tasks.**

1. *Picture Emotional Stroop Task (median emotional interference scores, seconds)*

|  | **UK** | |  | **Hong Kong** | | **Hong Kong migrants to UK** | | | | | **UK migrants to Hong Kong** | | | | |  |
| --- | --- | --- | --- | --- | --- | --- | --- | --- | --- | --- | --- | --- | --- | --- | --- | --- |
|  | (n=36) | 95% CI | | (n=39) | 95% CI | | ST  (n=37) | 95% CI | LT  (n=31) | 95% CI | | ST  (n=31) | 95% CI | LT  (n=28) | 95% CI | |
| Happy | 0.33 (0.96) | [-.08, .61] | | 0.05 (0.97) | [-.21, .44] | | 0.02 (1.06) | [-.26, .38] | 0.08 (1.01) | [-.15, .61] | | 9.29 (2.03) | [8.62, 10.18] | 10.06 (1.97) | [8.68, 10.66] | |
| Sad | -0.04 (0.78) | [-.50, .29] | | 0.06 (1.32) | [-.25, .50] | | 0.01 (0.65) | [-.30, .36] | 0.21 (1.08) | [-.12, .66] | | 9.24 (1.65) | [8.62, 10.15] | 9.92 (1.94) | [8.46, 10.39] | |
| Fear | 0.12 (0.10) | [-.27, .43] | | 0.16 (0.99) | [-.14, .53] | | 0.11 (0.87) | [-.19, .45] | 0.24 (1.12) | [-.02, .74] | | 9.11 (1.72) | [8.40, 9.90] | 9.90 (1.85) | [8.75, 10.65] | |
| Anger | 0.25 (0.79) | [-.07, .59] | | -.03 (1.04) | [-.36, .28] | | -0.10 (0.77) | [-.38, .21] | 0.12 (1.01) | [-15, .55] | | 9.48 (2.20) | [8.74, 10.34] | 10.08 (1.90) | [8.67, 10.69] | |

1. *Picture Attentional Probe Task (reaction time difference score: mean neutral minus mean emotional trial, msec)*

|  | **UK** |  | **Hong Kong** | | **Hong Kong migrants to UK** | | | | **UK migrants to Hong Kong** | | | |
| --- | --- | --- | --- | --- | --- | --- | --- | --- | --- | --- | --- | --- |
|  | (n=36) | 95% CI | (n=39) | 95% CI | ST  (n=37) | 95% CI | LT  (n=31) | 95% CI | ST  (n=31) | 95% CI | LT  (n=28) | 95% CI |
| Happy | 2.20 (32.56) | [-10.27, 12.78] | -2.17 (31.72) | [-12.31, 9.72] | -13.53 (34.00) | [-25.26, 2.43] | -.48 (41.87) | [-12.32, 14.68] | 7.80 (38.11) | [-5.58, 19.53] | 13.80 (33.34) | [-3.90, 27.49] |
| Sad | 1.76 (29.87) | [-8.05, 12.58] | 2.74 (27.62) | [-7.58, 12.13] | 2.14 (27.69) | [-7.50, 12.15] | 2.49 (35.16) | [-6.19, 17.03] | 11.49 (35.51) | [-1.50, 22.13] | 6.32 (32.71) | [-6.57, 22.98] |
| Fear | 7.50 (31.42) | [-4.46, 16.42] | -4.41 (26.99) | [-12.98, 6.98] | 5.83 (34.46) | [-5.25, 16.77] | 4.13 (43.75) | [-5.72, 20.32] | 5.55 (32.32) | [-10.62, 12.95] | -7.98 (37.76) | [-19.88, 9.60] |
| Anger | -5.90 (33.88) | [-15.90, 6.53] | 1.39 (28.71) | [-10.45, 10.98] | 17.84 (33.56) | [7.57, 28.70] | 2.03 (32.88) | [-7.07, 17.90] | 4.10 (34.46) | [-9.29, 14.73] | 5.85 (28.47) | [-9.46, 20.57] |

*Notes:* CI= confidence interval. ST= Short-term. LT= Long-term. UK = United Kingdom
